# Evaluating Barriers and Opportunities in Delivering High-Quality Oncology Care in a Resource-Limited Setting Using a Comprehensive Needs Assessment Tool

**DOI:** 10.1200/JGO.18.00125

**Published:** 2018-12-11

**Authors:** Chika R. Nwachukwu, Omobola Mudasiru, Lynn Million, Shruti Sheth, Hope Qamoos, Joseph O. Onah, Anita Okemini, Mojisola Rhodes, Michele Barry, Adekunbiola A. Banjo, Muhameed Habeebu, Tajudeen A. Olasinde, Ami S. Bhatt

**Affiliations:** ^1111^**Chika R. Nwachukwu**, **Lynn Million**, **Shruti Sheth**, **Hope Qamoos**, and **Ami S. Bhatt**, Stanford University School of Medicine; **Michele Barry,** Center for Innovation in Global Health, Stanford University, Stanford; **Omobola Mudasiru**, University of California Berkley, Berkeley, CA; **Joseph O. Onah**, **Anita Okemini**, and **Mojisola Rhodes**, Clinton Health Access Initiative, Nigeria Country Office; **Adekunbiola A. Banjo** and **Muhameed Habeebu**, Lagos University Teaching Hospital, Lagos; **Anita Okemini**, ONE Campaign, Nigeria Country Office, Abuja; and **Tajudeen A. Olasinde**, Amadu Bello University Teaching Hospital, Zaira, Nigeria.

## Abstract

**Purpose:**

Despite recognition of both the growing cancer burden in low- and middle-income countries and the disproportionately high mortality rates in these settings, delivery of high-quality cancer care remains a challenge. The disparities in cancer care outcomes for many geographic regions result from barriers that are likely complex and understudied. This study describes the development and use of a streamlined needs assessment questionnaire (NAQ) to understand the barriers to providing quality cancer care, identifies areas for improvement, and formulates recommendations for implementation.

**Methods:**

Using a comprehensive NAQ, in-depth interviews were conducted with 17 hospital staff involved in cancer care at two teaching hospitals in Nigeria. Data were analyzed using content analysis and organized into a framework with preset codes and emergent codes, where applicable.

**Results:**

Data from the interviews were organized into six broad themes: staff, stuff, system, space, lack of palliative care, and provider bias, with key barriers within themes including: financial, infrastructural, lack of awareness, limited human capacity resources, lack of palliative care, and provider perspective on patient-related barriers to cancer care. Specific solutions based on ability to reasonably implement were subcategorized into short-, medium-, and long-term goals.

**Conclusion:**

This study provides a framework for a streamlined initial needs assessment and a unique discussion on the barriers to high-quality oncology care that are prevalent in resource-constrained settings. We report the feasibility of collecting and organizing data using a streamlined NAQ and provide a thorough and in-depth understanding of the challenges in this setting. Knowledge gained from the assessments will inform steps to improve oncology cancer in these settings.

## INTRODUCTION

In contrast to global trends, the cancer mortality rates in low- and middle-income countries (LMICs) is rising.^1^ Although the ratio of cancer incidence to mortality in Africa is approximately 0.7, it is 0.36 and 0.46 in the United States and the European Union, respectively.^2^ The subtext for these disparities in LMICs can be explained by poverty, lack of high-quality health and cancer care, and limited preventive programs.^3-5^ In addressing the unique challenges faced by LMICs, an assessment of the existing system is the first step toward improving cancer care. For example, detailed assessments in Angola resulted in the development of actionable steps toward creating functioning cancer units and in Tanzania led to the implementation of a pediatric palliative care program.^6,7^

In Nigeria, a West African LMIC (Table 1 summarizes statistics), > 70% of its estimated 100,000 cancer diagnoses result in death annually. Although eight public tertiary teaching hospitals offer comprehensive cancer care, mortality remains high because of advanced disease at presentation, inadequate infrastructure to provide cancer treatment, limited access to systemic therapies, high costs of treatment, overworked staff, and lack of education and screening programs.^3,8,9^

**Table 1:**
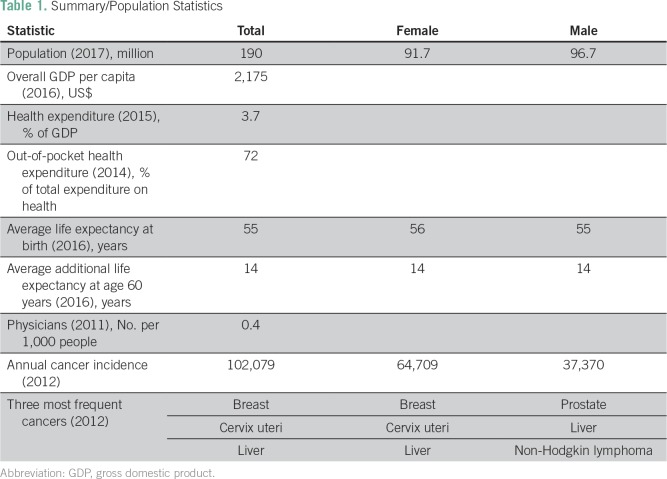
Summary/Population Statistics

In the published literature on cancer care in Nigeria, the focus is mainly on selected barriers associated with cancer care rather than comprehensive evaluations, which could inform our understanding of context-specific needs and facilitate development of region-specific cancer solutions. Therefore, a more comprehensive approach is required to capture the needs of the region. Furthermore, as improving cancer care becomes a priority in more LMICs, easily replicable and adaptable methods for needs assessment will ensure that the designed interventions, improvements, and solutions are well suited for a given region.

To understand these complex barriers to cancer care in Nigeria in more detail, this study used a comprehensive needs assessment questionnaire (NAQ) and a multidisciplinary approach to: collect objective data on barriers to cancer care, organize the findings into a previously defined framework that addresses health care issues in resource-constrained settings, and identify potential solutions to overcome said barriers.

## METHODS

This study was conducted in collaboration with six institutions: the American Cancer Society (ACS), the Clinton Health Access Initiative (CHAI), Stanford University, the nonprofit Global Oncology (GO), and two Nigerian teaching hospitals: Lagos University Teaching Hospital (LUTH) in Lagos and Ahmadu Bello University Teaching Hospital (ABUTH) in Zaria.

### Phase I: Preparation and Development of the NAQ

From May to October 2016, faculty at LUTH and ABUTH were matched with similar experts from Stanford University to define the goals of the collaboration and for Stanford faculty to gather information for use in developing the questionnaire. The NAQ was adapted from GO and modified by members of the Stanford Global Oncology Working Group and local CHAI representatives in Nigeria to ensure that it captured relevant information specific to the Nigerian population. The NAQ (Data Supplement) was divided into two sections: cancer assessment and human capacity. Focusing on various aspects of cancer management, the cancer assessment section included 53 broad questions and 111 subquestions over six thematic areas ranging from summary and health status of the population to barriers/challenges to cancer care. The human capacity section included six broad questions and 24 subquestions ranging from staff to education. Its adaptive design allowed respondents to answer only questions that pertained to their specialty.

### Phase II: In-Country Interviews Using the NAQ—Assessment of the State of Cancer Care

In February 2017, five Stanford medical professionals (four physicians and one registered nurse) traveled to Nigeria to conduct in-person interviews with the ABUTH and LUTH teams using the NAQ over a 4-day period. There were a total of 17 participants from LUTH and ABUTH, including physicians, pharmacists, and nurses. For most sessions, one moderator asked the questions while a second moderator took detailed notes. Each session lasted 4 to 6 hours.

The interview notes were analyzed using a content-analysis method to derive codes.^10^ Using this method, the responses were first sorted by discussion topics. Next, responses were categorized, and general themes were identified and then summarized. Themes considered distinct from the discussion topics emerged and were then coded to fit into preset codes and emergent codes if necessary.^11^ Preset themes were based on four key components to health care delivery proposed by Boozary et al^12^ for use in resource-poor settings (ie, the four Ss): staff, stuff, systems, and space (Table 2).

**Table 2:**
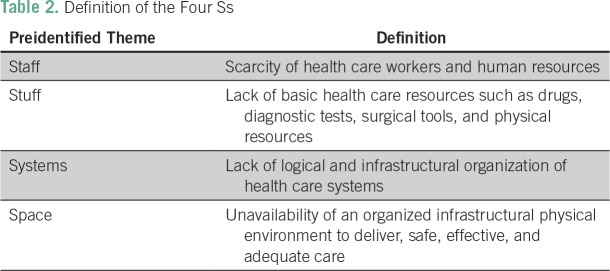
Definition of the Four Ss

Two researchers independently reviewed the notes and confirmed the validity of the themes. Theme saturation was considered attained when no new ideas or themes emerged. Finally, quotations that illustrated themes were selected and anonymized.

## RESULTS

Table 3 lists specific details about LUTH/ABUTH hospitals, including catchment areas, available resources, and staff. Analysis of the NAQ allowed coding of the interview notes into staff, stuff, systems, and space. Two additional themes arose that did not fit into these four broad themes: palliative care and patient-related barriers. Table 4 lists recommendations, which were generated for each theme, as potential solutions to strengthen delivery of cancer care in Nigeria (Data Supplement).

**Table 3:**
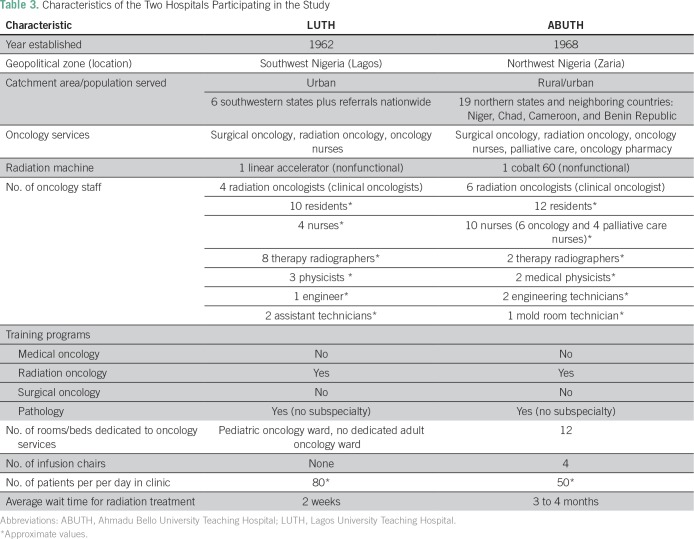
Characteristics of the Two Hospitals Participating in the Study

**Table 4:**
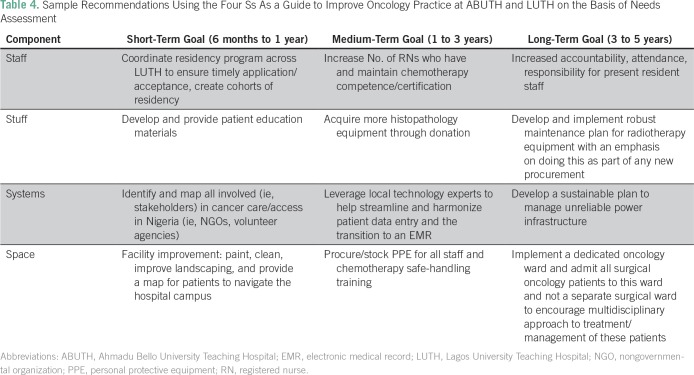
Sample Recommendations Using the Four Ss As a Guide to Improve Oncology Practice at ABUTH and LUTH on the Basis of Needs Assessment

### 

#### Staff.

A key deficiency was identified as limited human capacity. As one participant stated, “there is an increase in the burden of patients and [we] need more staff…. [There is] not enough support staff; we need to triple [the] number of staff” to adequately deliver high-quality care to patients with cancer. Participants generally reported feeling overburdened because of the high volume of patients. For example, ABUTH physicians consulted and treated approximately more than 50 patients with cancer per day without supporting staff and reported placing intravenous lines and reconstituting/administering chemotherapy, further limiting time spent with patients and contributing to work overburden.

A second major deficiency identified was the need for continuing medical education, access to relevant medical literature, expert opinions on challenging cases, and specialty training. For example, because of limitations in specialty training, LUTH providers reported relying on laparotomy procedures instead of minimally invasive surgical techniques, which resulted in the excessive use of more radical procedures.

#### Stuff.

Limitations seen in both hospitals included a lack of diagnostic radiology machines, absence of standardized treatment protocols for management decisions, and limited access to WHO essential medicines, including chemotherapy and other targeted therapies, because of their prohibitive costs. Furthermore, both institutions reported having one overworked radiation therapy treatment machine, delivering radiation therapy to approximately 80 to 140 patients per day. The linear accelerator machine at LUTH was nonfunctional, and the cobalt-60 at ABUTH, while functional at the time of the assessment, was at best intermittently functional. Machines remained nonfunctional for days to weeks because of unreliable maintenance or unavailable parts or repair services, and patients either did not receive treatment or traveled to other hospitals within or outside of the country, if financially possible. Another limitation emphasized included lack of patient access to cancer screening/prevention programs. For example, LUTH noted cervical cancer screening was available only to HIV-positive patients.

#### Systems.

The assessment highlighted several concerns including the lack of a national cancer registry or high-quality population-based registry, lack of cancer awareness/advocacy campaigns at the national level, inconsistent electricity/power supply, and limited information technology (IT) support. Both institutions used hospital-based cancer registries, which can fail to capture all treatment and follow-up information. Of note, follow-up data were rarely documented; as a member from the LUTH team stated, “patients are easily lost to follow-up; [only a] few patients who are motivated or who make good teaching cases are followed.” Another participant noted the need to transition from manual to electronic medical records: “[We] need improved medical records; currently [we are] using manual record keeping. [The] goal would be to use an electronic or computerized system to improve ability to do research” and ultimately aid in the advancement of patient care. Furthermore, both institutions also reported that in addition to limited governmental funding, cancer was not a major national health priority. For instance, participants reported that the National Health Insurance Scheme was implemented by the Nigerian Federal Ministry of Health (FMOH) in 2005 with the aim of improving affordable access to health care; however, only those employed in the federal sector (ie, < 5% of the working population) are enrolled, and only basic services (ie, limited oncology services) are covered.

#### Space.

Key areas for improvement included developing better-organized facilities (eg, designated patient waiting areas and maps/signs to assist patients in navigating through the hospital). One participant noted that “[we need] to revive the existing space” and that limited space represented one of the major barriers for individuals in hospitals. In addition, participants reported limited accommodation options for families who travel a significant distance to care for hospitalized patients. Furthermore, ABUTH reported no dedicated oncology unit, whereas LUTH reported limited oncology beds and inefficient outpatient chemotherapy delivery units. Cabinet space to safely store chemotherapy drug, and personal protective equipment often needed for mixing chemotherapy drugs were lacking.

#### Emergent themes.

Two additional themes emerged from the analysis but did not fit neatly into the preset themes. The first involved palliative care, specifically the lack of trained specialists in pain management and/or psychosocial counseling for patients. Physicians at ABUTH reported a fairly developed palliative care program, which engaged both spiritual and medical leadership but was inadequately staffed. LUTH had no active palliative care program or hospice services. Participants acknowledged the importance and need for these services; as one participant advised, “palliative care starts at…diagnosis; even though you cannot cure them, you can improve their quality of life.”

The second theme focused on the health care provider’s perspective on patient-related barriers to cancer care: structural-level and individual-level barriers. Structural-level barriers included the absence of a central patient referral system, limited health awareness programs/campaigns, unavailability of diagnostic machines for screening, long wait times, lack of specific guidelines for follow-up after patients have begun treatment, and overall high cost of health care services. Individual-level barriers were identified as limited patient knowledge, cultural beliefs (attitude toward mortality, no equivalent for cancer in the patient’s language, and use of traditional/faith healers), distrust of health care providers, and stigma of being diagnosed with cancer (generally seen as a death sentence).

## DISCUSSION

This study details the efforts of a nongovernmental agency, an academic institution, and partners in LMICs to identify barriers to quality cancer care in Nigeria.^13-15^ Using the NAQ, the data were organized with the four Ss as a conceptual framework to formulate solutions for short- and long-term improvements. The results of the study corroborated previously identified barriers in resource-poor settings.^3,5,12,16,17^ The study also identified additional themes including the lack of palliative care^18,19^ and the health care provider’s perspective on patient-related barriers to cancer care.

Increasing the capacity to have well-trained staff available at all levels is one key step in improving care outcomes.^3,4,17^ A short- and medium-term recommendation to decrease workload and ease physician overburden was the recruitment and training of support personnel including nurses, community health care workers, and other ancillary staff to allow health care providers to function effectively and efficiently. Specifically, using the Oncology Nursing Society curriculum, a Stanford registered nurse designed a weeklong training to certify Nigerian oncology nurses on core competencies necessary to safely administer chemotherapy. To date, a total of six nurses from ABUTH and LUTH have successfully completed the training and are Oncology Nursing Society certified. To facilitate distant learning where hands-on training is not feasible, full access for all staff to current and other online resources was recommended. The Stanford team facilitated yearlong access to the online medical education resource Up-to-date, arranged through the Global Health Delivery group. To foster collaborative work between specialties and prevent fragmented patient care, another short-term recommendation was the development of a multidisciplinary tumor board (MDC-TB), which was adopted by both institutions. A mock MDC-TB was designed to encourage collaboration between different oncology disciplines and standardize patient care and management through discussion, allowing for incorporation of peer-reviewed literature and adaptation of National Comprehensive Cancer Network guidelines. The short-term goal of increasing the number of trainees subspecializing in oncology was met by increasing their exposure to oncology specialties through the MDC-TB, and approval for a surgical oncology training program is being sought by LUTH.

One way to improve access to chemotherapy and WHO essential drugs^20,21^ is to develop a regionally coordinated purchasing arrangement. This would decrease the high and varying purchase prices of common chemotherapies, serve to stabilize the buyer’s bargaining power, and ensure that the necessary drugs are readily available. The long history of negotiation by CHAI in the drug supply chain and purchasing arenas for HIV drugs allowed it to add cancer-related therapies. In June 2017, ACS and CHAI announced a successful negotiation with Pfizer and Cipla to expand access to 16 essential cancer treatment medications, including chemotherapies, to Nigeria and five other sub-Saharan African countries. Additionally, the hospital/government is gaining traction in upgrading infrastructure at both hospitals; both ABUTH and LUTH are expecting new radiotherapy machines on the basis of ongoing discussions with two linear accelerator companies.

The development of a national cancer control plan (NCCP) has been reported as a critical step to improving care in LMICs.^3^ Cancer care was missing from the national health plan from 2014 to 2017. Incidentally, the 2018 to 2022 NCCP was in development at the time of this study, and recommendations from this study were incorporated into the plan, which officially launched in April 2018. In addition to an NCCP, improved cancer registries are needed to create a repository for studying cancers specific to the region to better understand risk factors, biology, and other factors that can improve outcomes. Although the Nigerian National System of Cancer Registry exists, and all cancer registries have the WHO/International Agency for Research on Cancer–designed software for data management, the absence of a central coding system has led to poor data quality. In addition, registry staff are still not comfortable using the software. One short- to medium-term recommendation was to engage with local IT professionals with the goal of leveraging resources and skills to create an electronic medical record system and improve hospital-based cancer registries. With the help of CHAI, the Stanford team and staff from LUTH liaised with local IT specialists, which provided a platform to communicate the technical challenges associated with patient management and potential benefits of an electronic medical record and functional cancer registry. Furthermore, to standardize patient care, the FMOH is in the process of endorsing the National Comprehensive Cancer Network treatment guidelines adapted for sub-Saharan Africa.

Facility design of a hospital is important for patient and health worker safety,^22^ and several recommendations were made to improve the overall patient experience, including: making physical changes to the building (cleaning, painting, and creating maps to help visitors navigate through the hospital), developing an oncology unit to improve chemotherapy delivery, creating a larger pediatric oncology clinic area adjacent to the inpatient ward, adding more isolation rooms, and providing adequate storage and biosafety cabinets to prevent patient and provider injury. Although these goals will largely rely on support from the FMOH, provisions for dedicated chemotherapy wards in select hospitals with the intent of upgrading their standards and a bill on the establishment of a national institute for the prevention, control, and treatment of cancers are under way.

Last, palliative care services are integral to cancer care control efforts.^23^ At the time of this study, ABUTH had a system in place for palliative care, primarily staffed by oncology nurses and volunteer assistants, but it lacked managerial and financial support. LUTH did not have dedicated palliative care staff/program. One recommendation was to develop a program with assistance from Stanford palliative care physicians.

From the participants’ perspective, one major barrier to care is poverty, which limits the likelihood of seeking care and paying for appropriate and sustained therapies once care has been established. Currently, 70% of health care payments are made out of pocket.^24^ One consideration was to expand the National Health Insurance Scheme to cover more patients and services associated with cancer management, similar to methods implemented with some success in some middle-income countries.^25,26^

There are several inherent limitations to this study. Only two university teaching hospitals were surveyed, so responses may not be generalizable to other parts of the country or care at private institutions. The selection of these hospitals was based on their tertiary care status; they treat a large majority of the patients with cancer in their respective regions. Additionally, the number of participants recruited for this assessment was low. Time limitations and the intensive, in-depth nature of this initial assessment precluded inclusion of all staff. Another limitation of the study is the absence of the perspectives of patients and their families on the barriers to cancer care. Although the study did identify patient-related barriers, it occurred from the provider’s perspective.

This study describes the evaluation and deployment of a streamlined yet comprehensive NAQ to understand barriers to cancer care, developed through a multidisciplinary collaboration that uniquely formed a twinning relationship with two tertiary care hospitals in Nigeria. This report used a qualitative approach to analyze and organize data gathered into meaningful short-, medium-, and long-term recommendations. The NCCP (2018 to 2022) currently takes most of these recommendations into consideration; however, the time to completion remains largely unknown. The limited resources and funding available in LMICs add unforeseen constraints to the sustainability and feasibility of the proposed recommendations. Nevertheless, these findings were discussed in detail with both hospital administration and the FMOH, which recognize the complexities and necessary commitments. These initial steps toward an international collaboration are aimed at promoting high-quality cancer care in Nigeria. The future goals of the Stanford/ACS/CHAI/GO collaboration with LUTH and ABUTH include establishing a working group committed to conducting pathology-based research, developing guidelines for working with radiation therapy, and improving palliative care. We anticipate that this newly adapted NAQ will be a useful starting point for other efforts geared at improving cancer care provision in other resource-constrained settings.
